# Underwater paddling kinematics and hydrodynamics in a surface swimming duck versus a diving duck

**DOI:** 10.1242/jeb.249274

**Published:** 2025-04-30

**Authors:** Hagar Csillag, Gal Ribak

**Affiliations:** ^1^ School of Zoology, Faculty of Life Sciences, Tel Aviv University, Tel Aviv 6997801, Israel; ^2^ The Steinhardt Museum of Natural History, Israel National Center for Biodiversity Studies, Tel Aviv 6997801, Israel

**Keywords:** Locomotion, Foot propulsion, Submerged swimming, Buoyancy, Anatidae

## Abstract

Some duck species mostly swim on the water surface while others frequently dive underwater. We compared the paddling kinematics of mandarin ducks (*Axis galericulata*) that feed on the surface and diving ferruginous pochards (*Aythya nyroca*) that feed underwater. Both species were trained to perform the same horizontal, submerged swimming at 1 m depth in a controlled set-up. Mandarins used alternate foot paddling exclusively, while pochards varied their gait between alternate foot paddling and simultaneous paddling with both feet. Unlike mandarins, pochards swam with their body tilted at an angle that was negatively correlated with the swimming speed and limited their foot motion to a smaller arc. Hydrodynamic modeling revealed that lift generated by the webbed foot provided thrust to propel both duck species forward. However, mandarins' feet generated lift-based upthrust that interfered with the need to counter their buoyancy, while pochards directed the foot lift to provide vertical downthrust against their buoyancy. The relatively subtle differences in foot motion between the two species result in a substantial hydrodynamic effect that may hint at the kinematic changes required when transitioning from surface to submerged swimming in the evolution of foot-propelled diving waterfowl.

## Teaser

**Summary:** Diving ducks use paddling kinematics underwater that is more efficient for countering buoyancy compared with that used by dabbling ducks.

## INTRODUCTION

Diving in birds has evolved independently at least 14 times in the course of avian evolution (Tyler and Younger, 2022). The need for locomotion in both air and water results in conflicting biomechanical requirements (Lovvorn and Jones, 1994; Wilson et al., 1992), primarily associated with buoyancy and propulsion (Segesdi et al., 2024). Flying birds have a low body density due to their hollow bones, air sacs and air trapped in their plumage. This low body density results in high buoyancy in water, which facilitates resting and swimming on the water surface but resists diving by drawing the bird upward (Lovvorn and Jones, 1991; Stephenson, 1994; Wilson et al., 1992). Indeed, diving birds spend a substantial portion of the energy invested in diving just to stay submerged, especially during shallow dives (<2 m) in which buoyancy due to the air-filled body volumes is maximal (Butler, 2000; Lovvorn et al., 1991; Ribak et al., 2010; Richman and Lovvorn, 2008; Stephenson, 1994; Watanuki et al., 2003).

The second biomechanical conflict involves using wings for propulsion in both air and water. The two mediums differ in their density and viscosity, making big wings used for flight inefficient underwater and small wings used for swimming (e.g. penguin flippers) inefficient for flight (Elliott et al., 2013). Accordingly, underwater wing propulsion in flight-capable diving birds is typically executed with partially folded wings to reduce effective wing area (Johansson and Aldrin, 2002). Some diving bird species may use both wings and feet underwater (Heath et al., 2006; Lapsansky and Armstrong, 2022). However, many waterbirds avoid the second conflict by swimming only using their webbed feet (‘foot propulsion’) with their wings folded next to the body.

Paddling with webbed feet is considered to be a form of drag-based propulsion as during surface swimming the hydrodynamic drag acts in the opposite direction to the backward-moving webbed feet, propelling the body forward. When swimming horizontally underwater, the propulsive force needs to push the body both forward and down to resist the body's buoyancy. The paddling motion is done by moving the feet relative to the body (in the body frame of reference) but the hydrodynamic forces are generated from the motion of the feet relative to the water in the world reference frame (WRF), in which the body is moving at the swimming speed. During horizontal dives along the bottom in fast piscivorous diving birds such as cormorants, grebes and loons, the backwards velocity of the feet relative to the body combined with the forward swimming velocity of the body results in minor backwards (relative to the swimming direction) feet motion in the WRF (Clifton and Biewener, 2018; Johansson and Norberg, 2001, 2003; Ribak et al., 2004). Instead, the feet mostly move vertically and/or sideways relative to the horizontal swimming direction at modest angles of attack (AoA), utilizing hydrodynamic lift to generate forward thrust (Clifton and Biewener, 2018; Johansson and Norberg, 2000, 2001, 2003; Ribak et al., 2004). Lift acts perpendicular to the direction of feet motion. It provides a more energetically efficient form of propulsion than propulsion based on drag, especially at higher swimming speeds (Walker and Westneat, 2000). Consequently, semi-aquatic mammals display a trend of transitioning from drag-based to lift-based propulsion, with increasing levels of aquatic existence (Fish, 2001). The functional morphology and diverse anatomy of waterfowl feet and legs have been studied intensively (Clifton et al., 2018; Segesdi et al., 2024; Zeffer and Norberg, 2003; Zeffer et al., 2003) and evaluated with respect to the trade-off between terrestrial and aquatic locomotion (Biewener and Corning, 2001; Clifton et al., 2018; Provini et al., 2012; Taylor-Burt, 2020; Taylor-Burt and Biewener, 2020). However, a trend of transition from drag-based to lift-based foot propulsion in waterfowl species differing in their foraging on and below the surface has not been demonstrated hitherto.

The Anatidae family constitutes a large group of waterbirds classified according to behavior as diving ducks, which forage underwater, and dabbling ducks, which feed mostly on the water surface. All species belonging to this family swim using their webbed feet. Both diving ducks and dabbling ducks swim on the surface and can dive to escape aerial predators, but only diving ducks rely on frequent foraging dives in which efficient underwater swimming is required. Studies have attempted to relate body density and the volume of plumage air to the level of diving in swimming birds (Lovvorn and Jones, 1991; Stephenson, 1995; Wilson et al., 1992). However, the underwater paddling kinematics and hydrodynamics have not been compared between diving and surface swimming duck species.

Depending on their habitat, a number of diving duck species feed mostly on sessile benthic vegetation and invertebrates. When feeding on sessile benthic prey, these ducks often dive vertically from the surface to the bottom, (Kaseloo and Lovvorn, 2006; Lovvorn et al., 1991), but may need to move horizontally along the bottom to reach adjacent food patches (Lovvorn and Brooks, 2021). When at the bottom in shallow water, diving ducks must paddle continuously against their buoyancy in order to remain submerged, whereas their ascent back to the surface is passive, exploiting their buoyancy (Lovvorn et al., 1991). During station holding next to the bottom in a diving sea duck (*Bucephala islandica*), when the mean forward swimming speed is zero and the duck's body is oriented vertically, the webbed feet are moved with their area oriented perpendicular to the velocity of the feet, making propulsion predominantly drag based (Ribak et al., 2010). During slow vertical descent from the surface to the bottom in a diving duck (*Aythya nyroca*), propulsion was mostly drag based except for the last 20% of the power phase, which exploited the lift generated by the feet for forward thrust (Ribak and Gurka, 2023). It is unknown how the backwards motion of the foot (relative to the body) during horizontal submerged swimming interacts with the forward speed of the duck to exploit lift and/or drag as the hydrodynamic force propelling the body forward.

Here, we compared the paddling kinematics of two species of ducks with different feeding habits. Mandarin ducks (*Aix galericulata*) are dabbling ducks (subfamily: Anatinae) mostly feeding on vegetation on the water surface or pond banks. Ferruginous pochards (*Aythya nyroca*) are diving ducks (subfamily Aythyinae) mainly feeding on submerged vegetation reached by diving to the bottom of shallow ponds (Johnsgard, 2010). The two species were chosen based on their foraging habits, availability and similar body size. The phylogenetic relationships within Anatidae, including the position of the *Aix* genus within it, are not fully resolved. Recent analyses place mandarin ducks in the Cairinini, a sister group of all other Anatinae tribes (Liu et al., 2021). Consequently, it is not entirely clear to what extent evolutionary history might have played a role in differentiating swimming between the two duck species. We trained both species to dive and swim along the bottom of a custom-built pool, and their paddling was analyzed during straight horizontal submerged swimming. We hypothesized that: (1) pochards would display paddling kinematics that is more suitable for submerged swimming, including higher use of lift-based propulsion and a more vertical direction of the propulsive force to counter buoyancy while swimming horizontally; (2) increased swimming speed would alter the trajectory of the feet in the WRF, increasing the relative contribution of lift to forward thrust in both species; and (3) this effect of underwater swimming speed would be more pronounced in the lift-based propulsion of pochards, reflecting their preference for feeding and swimming below the surface.

## MATERIALS AND METHODS

### Birds

**Fig. 1.**
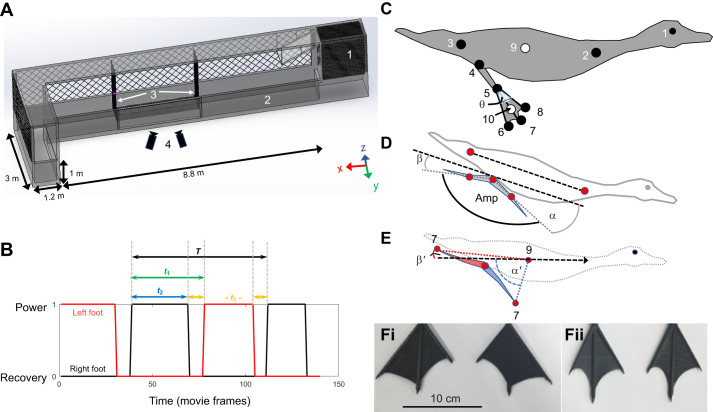
**Methods and analysis.** (A) Set-up for capturing the horizontal submerged swimming of the ducks. (1) Aviary, (2) straight section of the L-shaped pool, (3) dividers restricting the birds to swimming along the bottom, (4) high-speed cameras recording the ducks through a window. The front walls of the pool have been removed from the drawing to expose the interior. (B) Paddling gait analysis showing the definition of the cycle period (*T*), power phase duration (*t*_2_), duty cycle (*t*_2_/*T*), phase offset between feet (0.5−| *t*_1_/*T*−0.5|) and recovery overlap Σ*t*_3_/*T*. (C) The eight points on the duck (black circles) that were digitized in each movie frame: (1) eye on the side closer to the cameras, (2) base of the neck, (3) base of the tail, (4) tibia-tarsometatarsus (TMT) joint, (5) inter-digit joint (IDJ), and (6–8) tips of digits iv, iii and ii of the foot, respectively. Points 9 and 10 (white circles) correspond to the body center and center of foot force, respectively, that were found trigonometrically using other points (see below). A lighter area near point 5 depicts the inter-digit angle θ (Supplementary Materials and Methods). (D) Angles α, β and Amp used to describe the amplitude of the paddling motion in the sagittal plane. Red circles denote digitized points 2 and 3 on the body and 4 and 5 on the foot that were used to find these angles. (E) Angles α′ and β′ describe the position and orientation of the foot relative to the swimming direction (dashed arrow) upon power phase initiation (blue) and termination (red). (F) Foot planform. Shown are the 3D-printed models used for measuring the lift and drag coefficients of pochard (i) and mandarin (ii) feet (Supplementary Materials and Methods).

Six adult mandarin ducks, *Aix galericulata* (Linnaeus 1758) (two females, four males, mean±s.d. body mass 0.64±0.075 kg), and five adult ferruginous pochards, *Aythya nyroca* (Güldenstädt 1770) (two males, three females, 0.52±0.025 kg), participated in the experiments, which were performed in accordance with the ethical standards of Tel Aviv University Ethics Committee for Experiments with Animals (permit: AU-LS-IL-220110402) and the Israel Nature and Parks Authority (permit: 2023/43245). The shape of the webbed foot differs between the two duck species, with the foot of the pochards revealing less symmetry between the length of digits ii and iv (Fig. 1F). The planform shape and area of the feet were measured from images of the live birds or mounted museum specimens and used to estimate the added mass of the feet and to print 3D duck-foot models for force measurements (see Supplementary Materials and Methods and Fig. S1).

### Experimental set-up

The research system comprised a 13 m long, 1 m wide and 1 m deep, L-shaped pool with a small aviary to house the ducks at one end (Fig. 1A). The ducks were fed commercial hen food in the aviary. They were trained to swim to the other end of the pool where their preferred food (lettuce) was placed, and to return to the aviary in response to vocal signals. Next, we introduced a vertical divider into the pool and trained the ducks to dive under it in order to reach the other side. A second vertical divider was then added next to the first one. By gradually lowering the dividers and increasing the horizontal distance between them, while adding a horizontal mesh that prevented the birds from surfacing in between them, we trained the ducks to dive increasingly longer distances along the bottom. By the end of training, all the ducks swam on the surface towards the first divider, then dived vertically to the bottom, and swam horizontally along the bottom for 3 m before surfacing after the second divider at the other side of the obstruction. It took approximately 3 months to train the mandarins to perform the task while less than a week was required to train the pochards to perform the same task.

Two high-speed video cameras (Fastcam SA3, Phorton) operating at 250 frames s^−1^ filmed the diving ducks in lateral view through a window facing the middle of the 3 m long section during horizontal submerged swimming. The cameras were spatially calibrated using a two-points wand and the easyWand software (Theriault et al., 2014) to reconstruct the 3D positions of landmarks on the ducks' body and feet from the two camera views (Hedrick, 2008). The eye (point 1 in Fig. 1C) coordinates in the two camera views were used for fine tuning the preliminary results of the calibration algorithm (Theriault et al., 2014). The total (maximal) measurement error, evaluated by measuring known distances from the movies, was ±3 mm.

### Movie analysis

Field-of-view restrictions limited our analyses to a single paddling cycle per movie. It captured the ducks swimming 10–25 cm above the bottom of the pool. Each paddling cycle comprised a power phase and a recovery phase. We defined power phase initiation as the video frame showing the abducted digits of the webbed foot starting to move backward relative to the body. The video frame showing the end of the foot movement upward relative to the body marked power phase termination (and recovery phase initiation). For the paddling gait analysis, we binarily scored each movie frame as 1 if the foot was in power phase and 0 if the foot was in recovery phase. We then repeated the scoring for the contralateral foot. The cycle period (*T* in Fig. 1B) was defined from the initiation of the power phase of one foot to the next initiation of the power phase by the same foot. The paddling frequency was 1/*T*. The duty cycle of a single foot was defined as its power phase duration (*t*_2_ in Fig. 1B) divided by *T*. The time lag between initiation of the power phase in one foot and the initiation of the power phase in the contralateral foot was used to find the phase offset (Fig. 1B). A phase offset of 0.5 and 0 implies alternate and simultaneous paddling, respectively. The duration of sections within the paddling cycle where both feet were in the recovery phase (*t*_3_ in Fig. 1B) was summed and used to define the recovery overlap:
(1)

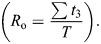
To analyze paddling kinematics, we focused only on the power phase of the foot closer to the cameras. The 3D position data were defined in a WRF aligned with the pool, where the *x*-axis is parallel to the length of the pool (positive end pointing in the swimming direction), the *y*-axis is parallel to the width of the pool, and the *z*-axis is the vertical axis (depth of the pool, pointing up). The pool geometry ensured that the swimming was straight and horizontal along the *x*-axis. The mean deviation (absolute values) from horizontal swimming (in the *xz* plane) of the sequences analyzed was only ±2.9 deg (s.d.=1.5 deg) and ±2.5 deg (s.d.=1.4 deg) for the mandarins and pochards, respectively. In the *xy* plane the deviations from the *x*-axis were ±5.6 deg (s.d.=2.5 deg) and ±4.8 deg (s.d.=3.4 deg). Using the Matlab code DLTdv5 (Hedrick, 2008), we digitized eight points on the duck's body and feet in each movie frame (points 1–8 in Fig. 1C). Two additional points were calculated from other points: the center of the body (point 9), defined half-way between points 2 and 3, and the center of hydrodynamic force of the foot (point 10), estimated to be at two-thirds the distance between points 5 and 7, based on the expected center of pressure for a triangular shape (Hoerner, 1985; Ribak et al., 2004).

From the positions of points 9 and 10, we calculated the instantaneous swimming and foot speeds, respectively, as in Rayner and Aldridge (1985). Averaging the instantaneous 3D body speed over the power phase duration gave the swimming speed. The longitudinal body axis was defined using points 2 and 3 (Fig. 1C). As the ducks' swimming was horizontal, with no visible body yaw or roll, we used point 9 to shift the data from the WRF to a coordinate system moving with the duck, where point 9 is the origin and *x*_b_, *y*_b_ and *z*_b_ are aligned with the *x-*, *y*- and *z*-axes in the pool-based WRF. We used this coordinate system to evaluate the foot's motion relative to the body. We measured the angle between the tarsus-metatarsus (the line connecting points 4 and 5) and the longitudinal body axis (Fig. 1C) in the *x*_b_*z*_b_ plane at the power phase initiation (α) and termination (β). The paddling amplitude (Amp) was defined as in Aigeldinger and Fish, (1995): Amp=180 deg−(α+β). In the WRF, we measured the angle between the *x*-axis (swimming direction) and the line connecting points 9 and 7 at the power phase initiation (α′) and termination (β′) (Fig. 1E). α′ represents the extent to which the foot was brought forward (relative to the swimming direction) at the end of the recovery phase. The foot's velocity vector and the orientation of digit iii (line between points 5 and 7) in the WRF were used to find the AoA of the foot in each video frame as in Ribak and Gurka (2023). Briefly, in each movie frame, the AoA is measured between the instantaneous velocity of point 10 (Fig. 1C) and digit iii in the plane normal to the span (i.e. neglecting the spanwise component of the flow). The angle is measured from the proximal edge of the digit (point 5) with oblique angles implying foot motion with the interdigit joint leading, whereas obtuse angles correspond to foot motion with the distal tip of the digit leading.

To compare the kinematics and instantaneous forces of power phases differing in duration, we normalized time within the power phase (*t*) of each movie by dividing it by the power phase duration (*t*_2_ in Fig. 1B) of the same movie, yielding a non-dimensional time scale of 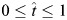. The kinematics or force data were then interpolated to 25 fixed time intervals (each 4% of the power phase duration) using cubic-spline.

### Biomechanical modeling of paddling hydrodynamics

We estimated the propulsive force generated by the feet based on the paddling kinematics using a simplified quasi-steady biomechanical model. The propulsive force at each instant of the power phase is a combination (vector addition) of instantaneous hydrodynamic lift, hydrodynamic drag and acceleration reaction (Ribak et al., 2004). Specifically, using the foot's instantaneous velocity (*U*) and AoA in the WRF, we estimated for each movie frame the instantaneous drag (*D*) and lift (*L*) as:
(2)

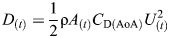
and
(3)

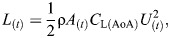
respectively, where the subscript (*t*) denotes that the calculated force changes over time, ρ is the density of freshwater (1000 kg m^−3^) and *A* is the webbed area of the foot in m^2^. In the calculation, we neglect the instantaneous contribution of foot rotation to the linear velocity 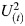 over the chord. *A* was allowed to change based on the instantaneous inter-digit angle, θ (Fig. 1A) in each movie frame (see Supplementary Materials and Methods and Fig. S2). *C*_D_ and *C*_L_ are the drag and lift coefficients, respectively, for a given AoA. They were determined empirically in a wind tunnel on the 3D-printed duck feet models (see Supplementary Materials and Methods and Fig. S1). The 3D-printed models were prepared for each duck species based on the morphological measurements extracted from the images of the feet.

The acceleration of the foot and the water surrounding it results in inertia. The acceleration reaction force due to this inertia (*F*_i_) is defined as:
(4)

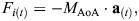
where *M*_AoA_ is the virtual mass of the foot for a specific AoA (foot mass+added mass of water accelerated with it; see Supplementary Materials and Methods), and **a** is the instantaneous acceleration vector of the foot calculated from the second time derivative of the positions of the foot (point 10) over time. We empirically measured the body density of mandarin duck carcasses using the volume displacement technique (Lovvorn and Jones, 1991) and estimated the buoyancy of the pochards based on published data (see Supplementary Materials and Methods and Table S1).

### Statistical analysis

Although all the ducks participated in the experiments, one female mandarin duck consistently used wing propulsion underwater (Movie 1) and was removed from the study. Because of uncertainty in distinguishing between some of the pochards in the movies, we treated each movie as a statistically independent swimming trial. The analysis of the paddling gait is based on 28 and 30 movies (paddling cycles) of mandarins and pochards, respectively. Of these, only 17 paddling cycles from 5 different mandarin ducks and 30 paddling cycles from 5 different pochards provided all the data points needed to extract the 3D kinematics of the foot. To compare between species, we used *t*-tests (two-tailed), or general linear models (GLMs) when accounting for covariation in the data. Non-parametric statistics (Mann–Whitney test) were used if normality could not be assumed (Shapiro–Wilk test). Similarly, parametric correlation coefficients (Pearson) and non-parametric correlation coefficients (Spearman) were used according to the normality test and denoted using the letters *r* and *R*, respectively. Unless otherwise stated all means are reported below ±s.d.

## RESULTS

### The paddling gait

**Fig. 2.**
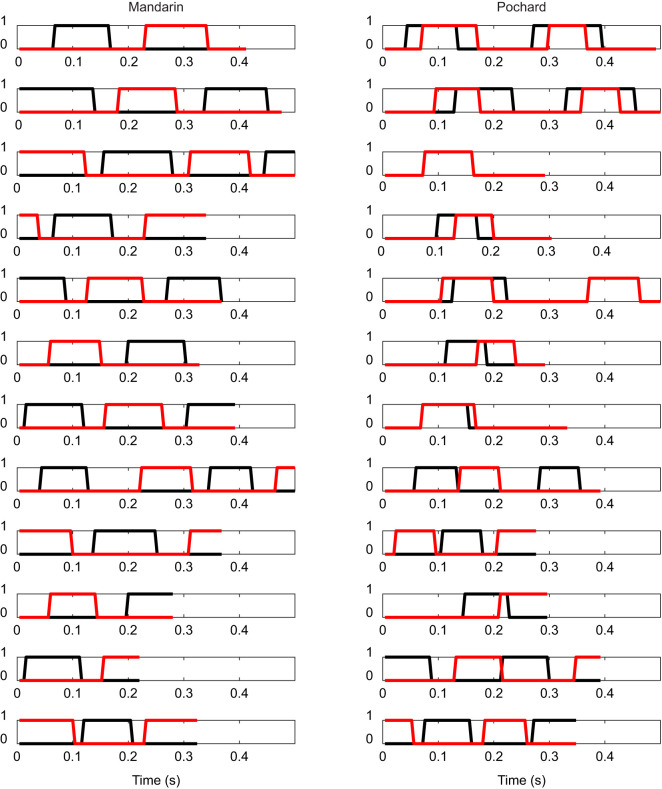
**Paddling gaits of submerged swimming ducks.** Each row represents a single movie, in which each frame was scored as 1 when the foot was engaged in a power phase and 0 when it was not. Red and black lines denote the scores for the contralateral feet as a function of time. Mandarins (left column) consistently used alternate paddling, while pochards (right column) varied their gait between alternate paddling and simultaneous paddling with both feet. Data from 12 randomly selected movies from each duck species (5 birds per species). The feet in the third pochard movie (from the top) fully overlap.

While swimming horizontally underwater (Movie 1), mandarin ducks used alternate paddling of the left and right feet exclusively, while pochards displayed a variable gait ranging between alternate paddling and simultaneous paddling with both feet, including various intermediate modes in which the power phases of the two feet only partially overlapped (Fig. 2). The mean phase offset was 0.45±0.043 in mandarins (*n*=28) and 0.26±0.167 in pochards (*n*=30). In both species, the power phase duration of the foot was shorter than its recovery phase. The mean duty cycle for a single foot (power phase duration/paddling cycle duration) was 0.32±0.053 in mandarins and 0.37±0.038 in pochards. Consequently, in both duck species and regardless of whether the gait was alternate or simultaneous, both feet were in the recovery phase during a part of the paddling cycle where no thrust could be generated. This recovery overlap (*R*_o_) was 35.7%±10.6% for mandarins and averaged 39.4±15.5% for all pochards with their variable paddling gate. There was no significant difference between the *R*_o_ of the two duck species (*t*-test, *P*=0.29).

### Modulation of paddling with swimming speed

**Fig. 3.**
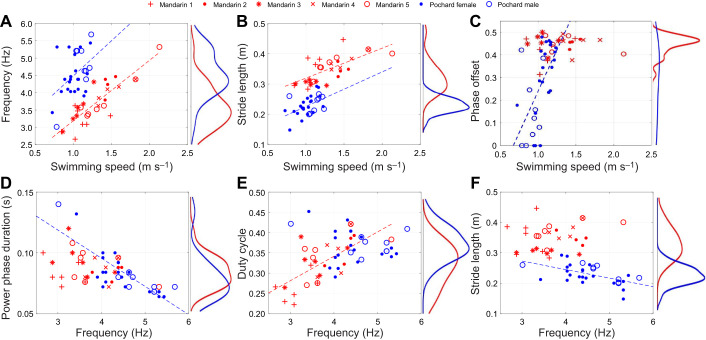
**Properties of the paddling gait.** Top: relationships between swimming speed and (A) paddling frequency, (B) stride length and (C) phase offset (0.5−|*t*_1_/*T*−0.5|; see Fig. 1B). Bottom: relationships between paddling frequency and (D) power phase duration, (E) duty cycle (*t*_2_/*T*; see Fig. 1B) and (F) stride length. Dashed lines denote statistically significant correlations using the same color code. Each data point represents a single paddling cycle. Open and filled blue circles denote male (2 birds) and female (3 birds) pochards, respectively; red symbols correspond to different individual birds within the mandarins (5 birds). Red (mandarins) and blue (pochards) curves to the right of each subplot depict the distribution (kernel density) of the dependent variable.

The swimming speed of mandarin ducks was significantly faster than that of pochards (1.28±0.28 versus 1.03±0.14 m s^−1^, *t*-test, *P*<0.001; Fig. 3). Both duck species increased paddling frequency when swimming faster (mandarin: *r*=0.85, *P*<0.001; pochards *r*=0.45, *P*=0.014), but pochards demonstrated a higher paddling frequency than mandarins for the same swimming speed (ANCOVA, *P*<0.001; Fig. 3A). Consequently, the stride length (distance traveled during a paddling cycle=swimming speed/paddling frequency) was significantly longer in mandarins (0.35±0.042 versus 0.23±0.031 m, GLM with swimming speed as a covariate, *P*<0.001; Fig. 3B). The variable phase offset observed in pochards was positively correlated with their mean swimming speed (*R*=0.62, *P*<0.001), with those pochards that swam faster tending towards alternate paddling (Fig. 3C). As the paddling frequency increased, the power phase duration remained unaffected in mandarins (*r*=−0.3, *P*=0.12), but tended to decrease in pochards (*R*=−0.78, *P*<0.001). In mandarins, the duty cycle increased with paddling frequency (*r*=0.67, *P*<0.001), while remaining unaffected by paddling frequency in pochards (*r*=−0.19, *P*=0.3; Fig. 3E). The stride length was unaffected by the paddling frequency in mandarins (*r*=0.18, *P*=0.35; Fig. 3F), but decreased with paddling frequency in pochards (*r*=−0.55, *P*=0.002).

### Body orientation

**Fig. 4.**
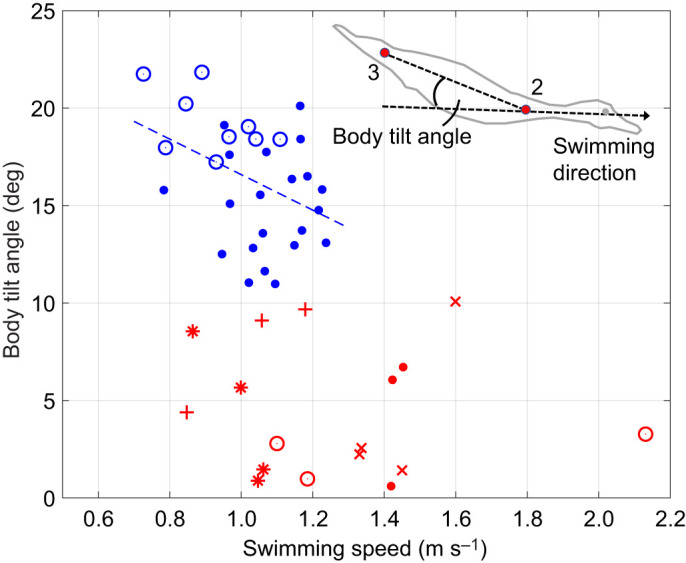
**Body tilt angle of swimming pochards and mandarins as a function of swimming speed.** Symbol shape and color are the same as in Fig. 3. Data are from 5 pochards (blue) and 5 mandarins (red). Points 2 and 3 correspond to digitized points in Fig. 1C.

Mandarins tended to swim with their body almost leveled parallel to the swimming direction, while pochards tilted their bodies head-down and tail-up so that their body's longitudinal axis tilted at a pitch angle of 16±3.1 deg with respect to the swimming direction (Fig. 4). The body tilt angle of the pochards (but not the mandarins) was negatively correlated with their swimming speed (pochards *r*=−0.53, *P*=0.002; mandarins *r*=−0.38, *P*=0.137). Neither species displayed pronounced body tilt fluctuations during the paddling cycle (Movie 1).

### Foot kinematics during the power phase

**Fig. 5.**
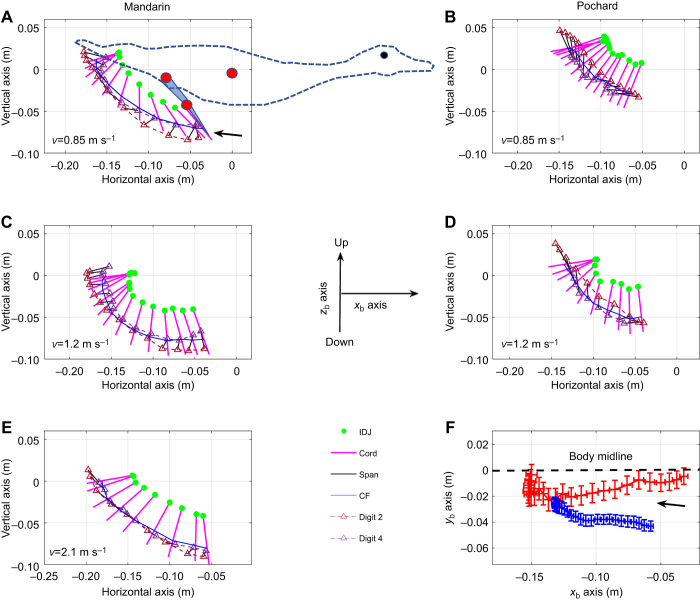
**Side view (sagittal plane) showing the trajectory and orientation of the foot relative to the body during the power phase.** See Fig. S3 for additional planes. Top, middle and bottom panels depict examples of low, medium and high swimming speeds (*v*) in mandarins (A,C,E) and pochards (B,D). Pochards did not reach the high swimming speed of 2 m s^−1^. Magenta lines depict the instantaneous position and orientation of digit iii (chord) relative to the center of the duck's body (0,0) as illustrated by the duck image in A. The image presents the duck upon power phase initiation, with red circles denoting digitized points 4, 5 and 9 from the movies. The remaining position data are shown at intervals of 8 ms (in the direction of the arrow) over the period of the power phase. Green circles depict the position of the inter-digit joint (IDJ, point 5). Blue lines trace the position of point 10 (center of foot force, CF) and triangles depict the tips of digits ii and iv. (F) Motion of the foot (point 10, see also Fig. S3) relative to the center of mass in the lateral (*y*_b_) axis during the power phase of mandarins (red) and pochards (blue). The black dashed line depicts the body's axis of lateral symmetry. Each point is the average of all paddling cycles at intervals of 4% of the power phase. The higher proximity of individual data points in the pochards is due to the same number of data points being used to illustrate the foot motion in the two species despite the pochards having a smaller translation in the *x*_b_ axis (compare A with B). The horizontal and vertical error bars depict the standard error of the mean position on the horizontal and lateral axes, respectively. Note the smaller paddling arc of pochards (e.g. in F, or compare A with B).

**Fig. 6.**
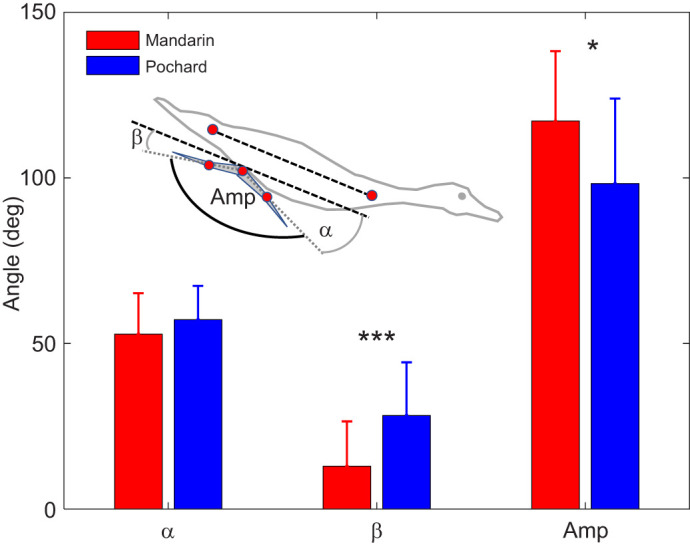
**Mandarins paddle at a larger amplitude.** The tarsus-metatarsus angle relative to the body's longitudinal axis upon power phase initiation (α) and termination (β) gives the amplitude (Amp) of the paddling arc angle as illustrated in the inset. Bars are means from *n*=14 (β, Amp) or 17 (α) mandarin paddling cycles (by 5 different birds) and 30 (α) or 29 (β, Amp) pochard paddling cycles (by 5 different birds). Error bars denote standard deviation. *P<0.05; ***P<0.001 in a *t*-test.

**Fig. 7.**
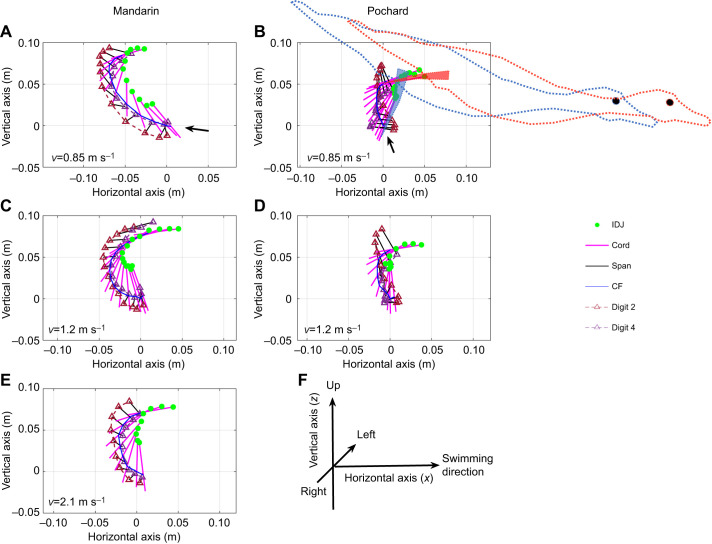
**Lateral views of foot trajectory in the world reference frame (WRF) during the power phase.** The origin of the coordinate system was set as the position of point 10 at power phase initiation. Top, middle and bottom panels depict examples (single power stroke) of low, medium and high swimming speed (*v*) in mandarins (A,C,E) and pochards (B,D). The highest swimming speed of pochards did not exceed 1.3 m s^−1^. Magenta lines depict instantaneous positions and orientations of digit iii (chord) at intervals of 8 ms (in the direction of the arrow) over the period of the power phase. Green circles depict the positions of the IDJ (point 5). Blue lines trace the position of point 10 (CF). Triangles depict the tips of digits ii and iv. Blue and red duck images in B illustrate the orientation of the foot and body of pochards upon power phase initiation and termination, respectively. (F) Definition of the horizontal and vertical axes in A–E.

The foot moved caudally relative to the body at the start of the power phase and dorsally towards the end in both duck species (Fig. 5). Motion of the foot in the lateral (*y*_b_) axis was minor in both species compared with that in the *x*- and *z*-axes. It mostly reflected the difference in foot planform between the two species (Fig. 5F; Fig. S3). The angle between the tarsus-metatarsus and the longitudinal body axis upon power phase initiation (α; Fig. 6) did not differ between the species (*t*-test, *P*=0.197). However, pochards terminated the power phase at a significantly larger β angle (*t*-test, *P*=0.004), resulting in a significantly smaller paddling arc (95±20.6 versus 117±21.1 deg, *t*-test, *P*=0.022; Figs 5 and 6). Within pochards, α, β and Amp did not differ (*t*-test, *P*>0.8 for all) between six paddling cycles showing the ducks swimming with simultaneous paddling and six paddling cycles showing the ducks swimming with alternate paddling. The similar angle α between mandarins and pochards combined with the larger body tilt angle of the pochards resulted in their not bringing their feet as far forward relative to the swimming direction in the WRF at the start of the power phase (Fig. 7). α′ (Fig. 1E) was positively correlated with swimming speed and was smaller in pochards (GLM with swimming speed as a covariate, pochards: α′=46.6±7.8 deg, mandarins α′=73.2±8.4 deg, *P*<0.001).

The ducks also differed in the orientation of the foot upon power phase initiation: in mandarins, the tip of the digits pointed forward whereas they pointed backwards in pochards (Fig. 7A versus B). Because of the shorter paddling arc and tilt of the body of pochards, both duck species ended the power stroke at low β′ angles that were negatively correlated with swimming speed The difference in β′ between ducks was statistically significant (GLM with swimming speed as a covariate, *P*=0.01) but amounted to only a few degrees **(**mandarins −1.7±5.1 deg, pochards 3.3±3.6 deg).

**Fig. 8.**
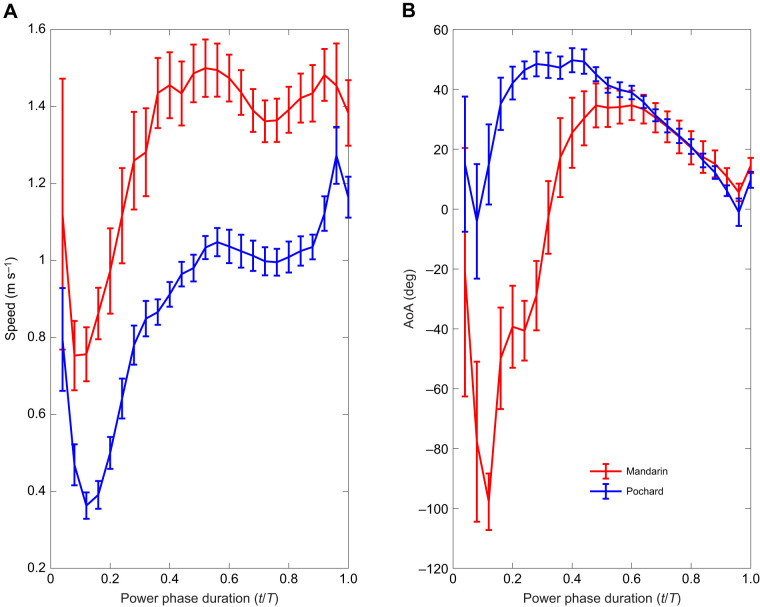
**Changes in foot velocity and angle of attack (AoA) during the power phase.** (A) Foot velocity is the speed of point 10 in a plane perpendicular to the foot span (in the WRF). (B) AoA is measured between the chord (digit iii) and foot velocity in the same plane. Curves present means of all the paddling cycles of mandarins (red, *n*=17 cycles from 5 birds) and pochards (blue, *n*=30 cycles from 5 birds). To average the data, the power duration was normalized to 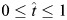 and interpolated using cubic-spline to fixed intervals (4% of the power phase duration). Error bars represent the standard error of the mean.

Combined with the forward movement of the body, the trajectory of the mandarin's foot in the WRF during the power phase was backward, then vertical (upward), and finally forward relative to the horizontal swimming direction. In pochards, the foot motion during the power phase in the same reference frame was mostly vertical, followed by motion of the foot in the swimming direction (Fig. 7). With the increase in forward swimming speed, the backward foot motion in mandarins diminished (Fig. 7E) and the foot moved upward and then forward, as in the pochards. These trajectory changes during the power phase affected the velocity of the foot in the WRF and the orientation (AoA) of the webbed area of the foot relative to this velocity (Fig. 8). In both species, the foot velocity increased rapidly during the first half of the power phase (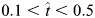), then remained at a relatively high value for the remaining of the power phase. Mean foot velocity in the plane perpendicular to the span was significantly higher in mandarins (1.3±0.23 versus 0.89±0.14 m s^−1^, *t*-test, *P*<0.001). Pochards had a low AoA during the first 10% of the power phase, which rapidly increased to ∼50 deg by 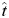
=0.2 and then gradually decreased to <10 deg by 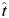
=0.96. In mandarins, the power phase started with the foot at a large (negative) AoA (∼−90 deg) that resulted from the tip of the digits pointing forward (in the swimming direction) at that instance (Fig. 7A,C) and having a backwards and downwards motion. The AoA then became positive as the foot rotated and reached a maximum of ∼40 deg only at 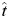
=0.44, and then matched the gradual decline to ∼10 deg observed in pochards (Fig. 8B).

### Estimated propulsive force

**Fig. 9.**
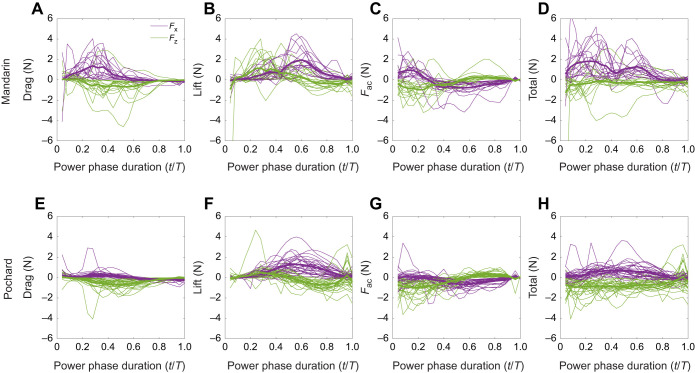
**Forces generated by the foot during the power phase.** (A,E) Drag, (B,F) lift, (C,G) acceleration reaction force (*F*_ac_) and (D,H) total force (vector sum of all three forces) for mandarins (top) and pochards (bottom). Each line represents the forces from a single power phase (mandarins: *n*=17 cycles from 5 birds; pochards: *n*=30 cycles from 5 birds). Purple lines denote the force component parallel to the swimming direction (*F_x_*, positive=thrust in the swimming direction). Green lines denote the vertical component of the force (*F_z_*, negative=downward directed force that can be used for opposing buoyancy).

The quasi-steady biomechanical model was used to relate the time-varying foot velocity and AoA to the instantaneous hydrodynamic forces generated by the foot (Fig. 9). The drag generated by the feet of both duck species had a downward (negative) vertical component that contributed to the birds' swimming horizontally by countering some of their positive buoyancy (Fig. 9A,E). In mandarins, foot drag also contributed to forward thrust during the first 50% of the power phase, when the foot moved backward in the WRF (Fig. 7A,C). In pochards, the contribution to forward thrust from drag was negligible (Fig. 9E).

Hydrodynamic lift generated during the last two-thirds of the power phase contributed substantially to both forward thrust and downward thrust in both duck species (Fig. 9B,F). In mandarins, a strong lift-based upthrust was also generated at the beginning of the power phase (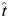
<0.4). Such upthrust at 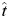 <0.4 was absent in pochards.

In mandarins, the acceleration reaction of the foot (Fig. 9C) created a strong forward and downward thrust upon power phase initiation, followed by equally strong backward, and weaker upward, directed forces at power phase termination. In pochards (Fig. 9G), the acceleration reaction during power phase initiation was mostly vertical (downward), and power phase termination resulted in upward and backward forces.

**Fig. 10.**
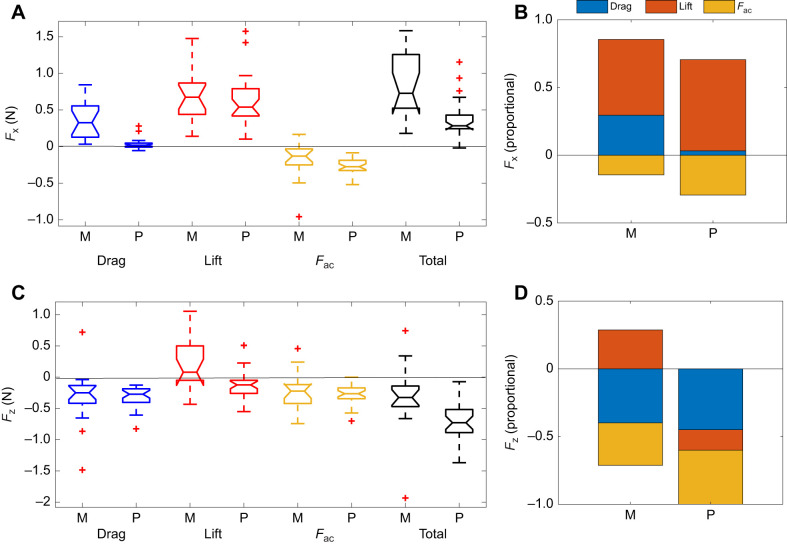
**Contribution of the three hydrodynamic forces to the total propulsive force.** Forces were averaged over the power phase duration of each movie and decomposed to the horizontal (*F_x_*, positive in the swimming direction; A,B) and vertical (*F_z_*, positive in the upwards direction; C,D) components. Boxplots in A and C depict the median across movies (horizontal line) with the bottom and top edges of the box indicating the 25th and 75th percentiles, respectively. Whiskers denote the maximum and minimum values not including outliers (red ‘+’). The data comprised 17 cycles from 5 mandarins and 30 cycles from 5 pochards. Stacked bar charts in B and D illustrate the relative contribution of each force to the total mean propulsive force in the same data. M, mandarins; P, pochards.

Combining the three forces (Fig. 9D,H) revealed that in mandarins both the vertical and horizontal force components presented distinct peaks within the power phase, whereas the total hydrodynamic force tended to distribute uniformly throughout the power phase in pochards. When the instantaneous forces were averaged over time for the entire power phase, the opposing acceleration reaction forces at power phase initiation and termination resulted in a net backward and downward force in both species (Fig. 10). The upthrust due to lift created upon power phase initiation in mandarins outmatched their downward directed lift created in the last two-thirds of the power phase, resulting in a net lift upthrust (Fig. 10C). The magnitude of the total propulsive force was similar in the two species, but its direction with respect to the swimming direction differed, with more of the force directed to resisting buoyancy in pochards.

The total force averaged over the power phase duration for one foot was 0.32±0.56 N and 0.71±0.27 N for the vertical axis (counter buoyancy) in mandarins and pochards, respectively (Fig. 10C). Combining the vertical force from two feet corresponded to 21±34.7% (minimum −48%, maximum 125%) and 65±24.5% (minimum 7%, maximum 124%) of the mean buoyancy of mandarins and pochards, respectively (buoyancy: mandarins 3.1±0.36 N pochards 2.2±0.11 N; see Table S1). On the horizontal axis, the mean forward thrust during the power phase of one foot was 0.9±0.46 N for mandarins and 0.4±0.25 N for pochards (Fig. 10A) with no and weak correlation between forward thrust and swimming speed in mandarins (*r*=0.29, *P*=0.42) and pochards (*r*=0.36, *P*=0.05), respectively.

The mean horizontal thrust due to foot drag during the power phase remained independent of the swimming speed in both duck species (*r*<0.09, *P*>0.61 in both cases), while the mean horizontal thrust due to foot lift increased with swimming speed in both species (mandarins: *r*=0.57, *P*=0.017; pochards: *R*=0.44, *P*=0.015; see Fig. S4). In both, the total downthrust increased with swimming speed (mandarins: *r*=−0.74, *P*<0.001; pochards: *r*=−0.49, *P*=0.006; Fig. S4).

## DISCUSSION

We hypothesized that the underwater paddling kinematics of pochards (diving ducks) would rely more heavily on lift-based propulsion and counter more of their buoyancy compared with that of mandarins (surface feeders). Indeed, we found distinct differences in paddling gait, foot kinematics and hydrodynamics between the two duck species when both performed the same type of horizontal submerged swimming. The observed differences support our hypothesis as discussed below.

### Differences in paddling kinematics and hydrodynamics

While foot movement relative to the body did not dramatically differ between the two duck species, the smaller paddling arc and larger body tilt in pochards were sufficient to change the trajectory and orientation of their foot in the WRF. The pochard's foot did not reach as far forward (in the swimming direction) in the recovery phase as that of the mandarin, and therefore moved mostly upward and then forward in the WRF during the power phase. Such foot trajectory generates drag with a large downward component and lift that is mostly directed forward and then down. Thus, in pochards, the force that propelled the body forward was almost entirely based on lift (Fig. 10B). In contrast, at the same swimming speeds, the mandarin foot had a backward motion relative to the swimming direction in the WRF and a different foot orientation at the beginning of the power phase. The drag force of the mandarin foot at that stage propels the body mostly forward while lift contributes upthrust, working with, rather than resisting, buoyancy. Later, when the mandarin foot starts to move vertically, lift is directed forward and then downward but the downward lift force at the end of the power phase is mostly canceled by the upward lift force at the beginning of the power phase, leaving drag and acceleration reaction as the only forces contributing to buoyancy resistance and to an overall lower vertical downthrust (Fig. 10C,D). At faster swimming speeds, the backward motion of the mandarin foot diminished in the WRF, but pochards were able to eliminate the upthrust during power phase initiation even at much lower swimming speeds. The improved ability of pochards to direct the propulsive force to resist their buoyancy at lower swimming speeds supports our hypothesis that their foot kinematics is better suited to submerged swimming than mandarins.

In diving cormorants, grebes and loons, the foot motions indicate use of lift for forward thrust coupled with a decrease in the contribution of foot drag for forward thrust (Clifton and Biewener, 2018; Johansson and Norberg, 2001, 2003; Ribak et al., 2004). Here, while both ducks used lift as the predominant source of forward propulsion, pochards relied on lift for forward propulsion exclusively (Fig. 10A). Lift-based propulsion is more efficient for fast swimmers because, as swimming speed increases, the backward paddling motion of the foot diminishes (e.g. compare Fig. 7A,C and E), limiting drag-based thrust production (Biewener and Patek, 2018; Vogel, 2013; Walker and Westneat, 2000). In contrast, in lift-based propulsion, the appendage is moved perpendicular to the swimming direction (here upward) and the lift forces generated may increase with the swimming speed. The negligible contribution of foot drag to forward thrust in pochards further contributes to the notion that their paddling kinematics is better suited to facilitate swimming underwater compared with mandarin ducks.

Both species generated a substantial vertical propulsive force that aided them in overcoming their buoyancy. The mean vertical force from the two feet was only equivalent to 21% and 65% of the estimated buoyancy in mandarins and pochards, respectively. These vertical propulsive forces are insufficient to counter the buoyancy of both duck species at 1 m depth because they correspond only to the mean force acting during the power phase while no propulsive force is generated during the foot's recovery phase. Our model may underestimate vertical forces generated during propulsion, or the birds may have additional adaptations to reduce their buoyancy. Cormorants swimming horizontally underwater counter some of their buoyancy by generating downward thrust from the lift generated by their tilted bodies (Ribak et al., 2004). Pochards with their tilted body might have done the same to supplement the downward thrust needed to overcome their buoyancy. A different explanation is expected for mandarins, which swam with their body leveled. We noted that they tended to swim with their wings folded less tightly against the body and posit that they used their higher swimming speed to generate additional downward thrust from lift generated by the loosely folded wings. However, we could not accurately corroborate this impression from the movies showing the ducks' lateral view. Such use of wing lift to counter the remaining buoyancy dictates fast swimming speeds and comes at a cost of higher body (and wing) drag, presumably explaining the 2.25-fold larger horizontal forces generated by mandarin paddling (Fig. 10A). The positive relationship between downward propulsive force and swimming speed (Fig. S4) might have also contributed to the higher swimming speeds of mandarins compared with pochards.

The quasi-steady model used here to estimate forces simplifies the feet as rigid hydrofoils and assumes that the unsteadiness in the flow can be represented by breaking down the motion into short intervals in which the flow is quasi-steady. Therefore, it does not presume to capture the full complexity of the flow during paddling; nevertheless, it is useful for comparing the paddling of the two species by giving a hydrodynamic interpretation to the observed changes in the kinematics. Inaccuracies due to the model assumptions should apply similarly to both duck species.

### Relationship between paddling hydrodynamics and swimming speed

We also hypothesized that swimming speed will affect the type of hydrodynamic forces generated by the feet to propel the body forward and that this effect may differ between the two species. We found that forward thrust due to lift generated by the feet increased with increasing swimming speed in both species but pochards utilized lift-based propulsion at lower swimming speeds than mandarins. In both species, the downthrust generated by the feet, helping to resist buoyancy, increased with swimming speed, while the forward thrust due to drag of the feet remained independent of the swimming speed (Fig. S4). Swimming speed changes the trajectory and AoA of the feet in the WRF. The contribution of lift to thrust in pochards comprised >70% of the forward thrust of the birds (Fig. 10B) and was generated during >50% of the power phase (Fig. 9F). In pochards descending the water column at a lower swimming speed (0.32 ms^−1^), the foot lift contributed to forward thrust only in the last 20% of the power phase (Ribak and Gurka, 2023). At even lower swimming speeds, other ducks (*B. islandica*) holding position next to the bottom (zero mean swimming speed) generated propulsive force that was reported to be entirely drag based (Ribak et al., 2010). Thus, higher swimming speed seems to play a role in increasing the contribution of lift to forward thrust in foot-propelled diving ducks.

### Alternate versus simultaneous paddling

We found that the total forward thrust averaged over the power phase did not increase with swimming speed in mandarins (that used alternate paddling) and had a marginal positive correlation with swimming speed in pochards that displayed a variable gait. In both species the expected increase in forward thrust to overcome the drag of a faster swimming body seems to come from increased paddling frequency (Fig. 3A), resulting in shortening of the recovery phase where propulsive forces are negligible. This shortening is evident as an increase in duty cycle with increased paddling frequency in mandarins (Fig. 3E). Increased paddling frequency was associated with a shortening of the power phase in pochards (Fig. 3D), leading to an unchanged duty cycle as paddling frequency increased.

Mandarins and pochards used alternate paddling exclusively while swimming on the surface (Fig. S5). Underwater, mandarins continued to use alternate paddling while pochards varied their gait between alternate and simultaneous paddling. A transition from alternate paddling on the surface to simultaneous paddling with both feet underwater has been reported in many diving waterbirds including cormorants, loons, grebes and diving ducks (Lovvorn, 1990; Townsend, 1909). In contrast, darters and mallards use alternate paddling both on the surface and underwater (Taylor-Burt, 2020; Townsend, 1909). The question ‘why do some diving birds transition from alternate paddling on the surface to simultaneous paddling while swimming underwater?’ was raised more than a century ago (Townsend, 1909) and has not yet been conclusively answered. Lovvorn (1990) proposed that the transition to simultaneous paddling underwater emerged from the need to generate larger forces to overcome both drag and buoyancy of the submerged body during the descent to the bottom. Swimming along the bottom may be less demanding than descending against buoyancy. Nevertheless, our observations on horizontal submerged swimming revealed that rather than transitioning to simultaneous paddling at greater swimming speeds, when the resistance of water to swimming is higher, the faster mandarins and pochards used alternate paddling, while the slowest swimming pochards demonstrated simultaneous paddling (Fig. 3C).

We suggest that the transition to simultaneous paddling during submerged swimming enables paddling at higher maximal frequencies. The duty cycle was <0.5 in both duck species, implying that the recovery phase is longer than the power phase. If the power phase duration is 1 and the recovery duration is 1+*x*, then, during simultaneous paddling, the theoretical paddling cycle duration (power+recovery) is 2+*x*, whereas, during alternate paddling, it is 2(1+*x*) with the longer recovery of the left and right foot being the limiting factors to shortening cycle duration. Consequently, the duty cycle of a foot in alternate paddling is 1/(2+2*x*). In mandarins, the mean duty cycle of one foot was 0.32. Solving for *x* and substituting gives a cycle period of 2+2*x*=3.125 and 2+*x*=2.56 power phase durations for alternate and simultaneous paddling, respectively. As frequency is the inverse of cycle period, mandarins can theoretically increase their paddling frequency by 22%, just by switching from alternate to simultaneous paddling. In practice, the recovery overlap in the alternate paddling gait of the mandarins made their paddling cycle duration <2+2*x*. In addition, pochards that displayed an intermediate gait between alternate and simultaneous paddling had cycle durations >2+*x*. Consequently, the mean recovery overlap was similar in the two duck species. Nevertheless, the basic principle that a longer recovery phase is a limiting factor for higher paddling frequency during alternate paddling remains valid. A longer recovery may be favored because it reduces the foot velocity during this phase and with it the backwards directed drag on the flexed foot that would slow the birds down. In contrast the power phase has to be fast (i.e. short) to generate sufficient propulsive force.

When mandarins increased their paddling frequency, their duty cycle increased, indicating that they had mostly reduced the recovery phase duration. They did display a moderate decrease in power phase duration with increasing paddling frequency (Fig. 3D) that increased the speed of the paddling foot, but did not contribute to increasing paddling frequency, which was limited by the longer recovery of the contralateral foot (duty cycle was always <0.5; Fig. 3E). In contrast, pochards reduced their power phase duration but not their duty cycle when paddling frequency increased, indicating that they decreased both the power and recovery phases simultaneously. While the mean power phase duration of the mandarins and pochards was similar (Fig. 3D), paddling frequency was higher in pochards. Thus, the flexible gait of pochards seems to enable a higher paddling frequency, whereas the mandarin ducks, which are mostly surface swimmers, seem to be constrained to the alternate paddling gait typical of surface swimming.

Simultaneous paddling may incur a high energetic cost in order to accelerate the body during the power phase to compensate for the deceleration of the body during the recovery phase. These cyclic accelerations can be eliminated through delivering thrust across the entire paddling cycle by using alternate paddling (Lovvorn, 1990). However, the mandarins' alternate paddling had a similar recovery overlap to that of the pochards, indicating that when duty cycle <0.5, alternate paddling is not a guarantee for continuous thrust delivery. Moreover, the propulsive force of mandarins displayed distinct peaks during the power phase of each foot, indicating that thrust production is not uniform even when paddling is alternate. These force peaks were absent in pochards (Fig. 9), contributing to their more uniform swimming speed when using alternate paddling. During their simultaneous paddling, smaller fluctuations about the mean swimming speed can be expected when the paddling frequency is higher for similar swimming speeds. Therefore, the flexible gait of pochards enabled them to benefit from the advantages of both simultaneous and alternate paddling. They were able to swim at higher paddling frequencies and distribute propulsive forces over a longer section of the paddling cycle.

### Foot morphology

We expected to find different hydrodynamic properties for the feet of the two species, but the different planforms had only a minor effect on the lift and drag coefficients measured in the wind tunnel (see Fig. S1). Pochards had a larger webbed foot area (mandarins 11.56 cm^2^, pochards 15.36 cm^2^) despite their slightly smaller body mass. Despite this difference in foot area, the mean (total) force generated per foot was similar between the two species because mandarins moved their feet faster in the WRF (Fig. 8A) while the larger foot area of pochards affected a larger water volume during paddling. Theoretical considerations indicate that for the same momentum transfer (from feet to water), Froude (propulsion) efficiency will be higher when large water volumes are moved at a low speed compared with small water volumes moved at higher speed (Vogel, 2013). The larger webbed foot area in pochards and slower motion of their feet suggest that their propulsion is more efficient.

Our study has described both the various kinematic adjustments made by pochards during horizontal submerged swimming and the submerged swimming of mandarin ducks, which seemed to swim underwater using the body orientation and paddling gait typical of surface swimming. The difference between the two species in foot kinematics and body tilt primarily resulted in differences in the velocity and orientation of the foot at the beginning of the power phase, with consequences for the magnitude and direction of the propulsive force. Despite their substantial hydrodynamic effect, these kinematic adjustments in body tilt and foot motion are relatively subtle, in the sense that they do not require the evolution of unique morphologies, likely facilitating the evolutionary transition in waterfowl from surface swimming to foot-propelled diving.

## Supplementary Material

10.1242/jexbio.249274_sup1 Supplementary information
