# Supplementary material for: Underwater paddling kinematics and hydrodynamics in a surface swimming duck versus a diving duck
Source: J Exp Biol. 2025 Apr 30;228(9):jeb249274. doi: 10.1242/jeb.249274 (PMC12079667; doi:10.1242/jeb.249274)
Supplement: Supplementary information [file jexbio-228-249274-s1.pdf]

Journal of Experimental Biology • Supplementary information

Suplementary Materials and Methods

Measurements of the lift and drag coefficients of the duck feet

The biomechanical model of foot propulsion presented in the main text requires input of the lift and drag coefficients of the feet as a function of their angle-of-attack (AoA). These coefficients were measured empirically on 3D duck feet models with the same planform shape as the feet of the pochards and mandarins. The 3D printed foot models (Fig. S1.1) were based on images of the live birds (n=6 for the mandarins, n=1 pochards), and a mounted museum specimen (of a ferruginous pochard) obtained from the Steinhardt Museum of Natural History. The webbed area of the feet was photographed from above when the ducks were standing. Two additional mounted specimens of pochards were used to measure the foot area and digit lengths and widths and the interdigit angles directly. From the images and measured data, we designed CAD models in Solidworks ©. Each digit in the CAD model had the average diameter measured from the ducks and the interdigital webbing had a thickness of 1 mm. The CAD models were SLA 3D-printed from Elegoo standard resin (Elgoo, China) and smoothed with fine sandpaper to remove printing surface imperfections. The force coefficients for the pochard foot and the measurement method are published elsewhere (Ribak & Gurka, 2023). For the force coefficients of the mandarins the methodology was identical. The foot model was tethered in the middle of the working section of a wind-tunnel and the fluid forces were directly measured in the horizontal (streamwise) and vertical (wall-normal) directions (drag and lift, respectively) using force transducers. Details of the wind-tunnel and force transducers setup can be found in Urca & Ribak (2021). We used dynamic similarity to maintain the Reynolds number of the measurement in air similar to the conditions of the foot during swimming through water: i.e., the 3D printed models were upscaled 2:1 relative to the real size and the wing speed was set to 6 ms^{-1}, resulting in a Reynolds number (based on the length of digit iii) of 40,000. The Reynolds numbers for the feet of the ducks during diving (measured from the movies) was 44,000-65,000. This slight difference can lead to a small underestimation and overestimation of the real foot's lift and drag coefficients, respectively (Jacobs & Sherman, 1939), thus keeping our study that seeks to

estimate the contribution of lift to paddling propulsion, conservative. The AoA was adjusted manually at increments of ~10° and verified to the nearest 1° in an image taken by a camera placed horizontally and perpendicular to the wind tunnel length, facing at the foot in side view. The forces at a given AoA were converted to the lift and drag coefficient as:

$$C_{D(AoA)} = \frac{2D_{(AoA)}}{\rho A U^2}$$

for the drag coefficient and

$$C_{L(AoA)} = \frac{2L_{(AoA)}}{\rho A U^2}$$

for the lift coefficient, with D and L corresponding to the measured drag and lift, respectively, for a given AoA, as measured by the force transducers. ρ is the density of air (1.2 kgm^{-3}), A is the planform area of the foot model and U is the wind speed in the wind tunnel (6 ms^{-1}). The relationship between the force coefficients and AoA was fitted with a 5th order polynomial equation that was used in the models to find the lift and drag coefficient for each duck species for each AoA (Fig. S1.1)

For the foot virtual mass of both species, we used the mean mass of the foot (3.4 gr) measured from two mandarin carcasses; and the added mass was estimated separately for each species from the planform of the above-mentioned foot images. In these pictures we measured the foot's chord at each 1 mm of the span. The added mass for each foot section (each 1 mm wide chord strip) at a specific AoA was estimated according to the elongated body theory (Lighthill, 1970) as described in Ribak et al. (2004). The added mass of all foot sections was summed to give the value for the entire foot. The resulting added mass was then corrected in each movie frame according to changes in the inter-digit angle observed in the movies (see below, Fig. S1.2)

Journal of Experimental Biology • Supplementary information

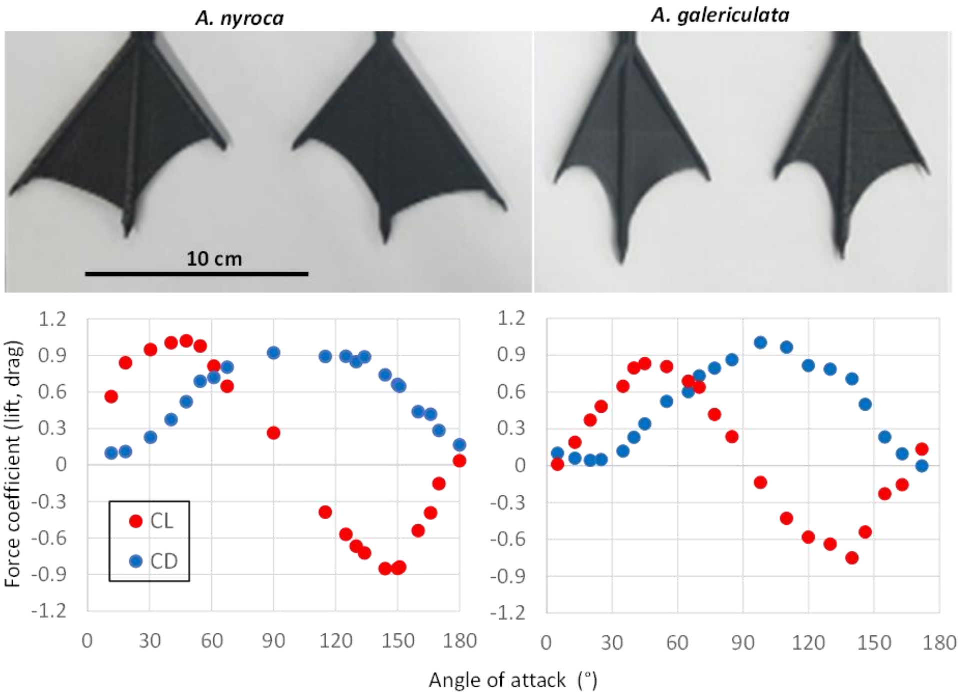

Fig. S1. The hydrodynamic performance of the duck foot models. The upper images show the 3D-printed duck foot models of the pochard (left) and mandarin (right). The lower panel presents the measured lift (red) and drag (blue) coefficients as a function of the models' AoA, as measured in the wind tunnel.

Foot area adjustment in the biomechanical model

The inter-digit angle θ changed during the power phase (Fig. S1.2) leading to an increase or decrease in the webbed area of the foot relative to the area measured from images of standing birds. We therefore used an instantaneous area for each movie frame ($A_{(t)}$) that was based on the measured $\theta_{(t)}$ at that video frame. Based on an approximation of the foot shape to a triangle the correction is:

$$A_{(t)} = A \frac{\tan\left(\frac{\theta_{(t)}}{2}\right)}{\tan\left(\frac{\theta}{2}\right)}$$

where A and θ are the area and inter-digit angle measured from the still images of the standing bids. $A_{(t)}$ was used in equations 1 and 2 of the main text to calculate lift and drag. Note that the

Journal of Experimental Biology • Supplementary information

correction is for the change in area of the foot but does not account for the change in aspect-ratio of the foot. The effect of small changes in aspect-ratio on the lift and drag coefficients are not expected to be significant since the feet are a low aspect-ratio hydrofoil to begin with. E.g., compare the similar lift and drag coefficients of the mandarin (aspect ratio =2, $\theta = 60°$) and pochards (aspect ratio =2.2, $\theta = 74°$) physical foot models in Fig. S1.1.

The change in aspect-ratio does affect the calculation of added mass for the foot. This is corrected by calculating the added mass from the local chords (c) extracted from the foot still images after correcting these chords with $\theta_{(t)}$:

$$c' = c\frac{\sin\left(\frac{\theta_{(t)}}{2}\right)}{\sin\left(\frac{\theta}{2}\right)}$$

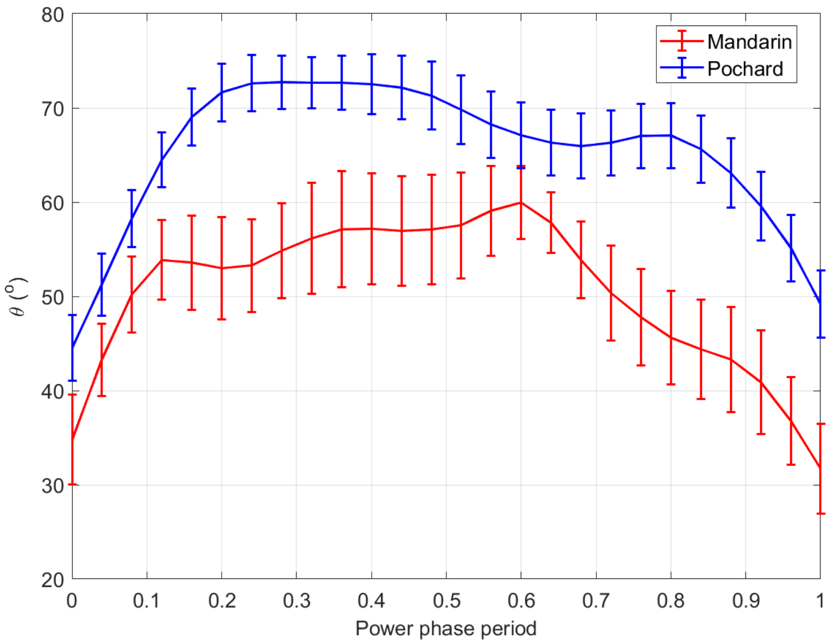

Fig. S2. The change in the inter-digit angle θ during the power phase. Red and blue lines correspond to average values from all movies of each species and error bars to the standard error. The average θ measured in images of the foot taken from standing live birds or mounted museum specimens was 60° and 74° in the mandarin and pochards, respectively.

Journal of Experimental Biology • Supplementary information

Body mass and buoyancy of the ducks used in the study

The buoyancy of the mandarins was estimated by measuring the body mass and volume of two carcasses as in Lovvorn and Jones (1991). Each carcass was strapped to a pole with adhesive tape and submerged head first for 60 seconds in a 1 m deep cylinder (diameter =15.6 cm) filled with water. The volume of water displaced by the submerged carcass was assessed from the rise of water level in the cylinder (measurement accuracy: ± 25 ml). The measured volume with the mass of the carcasses gave their density (ρ_{duck}); and buoyancy (B) was measured as:

$$B = (\rho_{water} - \rho_{duck})Vg,$$

where ρ_{water} is the density of freshwater (1,000 kgm^{-3}), V is the measured volume of the bird (in m^3), and g is gravitational acceleration (=9.8 ms^{-2}).

Due to concerns over the small sample size (n=2) and possible bias from measuring carcasses, we compared the measured mean density to published data for a different Aix species (*Aix sponsa*) measured by Lovvorn & Jones (1991) on live birds. The difference between the mean density of the two carcasses of *A. galericulata* and the mean density of live *A. sponsa* was only 5.5%. (Table S1). We therefore used the density of the carcasses to estimate the buoyancy of our live mandarin ducks (Table S1). For the pochards, we used the volume and mass of four other *Aythya* species measured by Lovvorn & Jones (1991). From their data we calculated the density for the four species (mean ρ_{duck} = 694.6, SD = 5.87,) and applied this mean density together with our known ducks' body mass to calculate the volume and buoyancy of our pochards (Table S1).

Journal of Experimental Biology • Supplementary information

Table S1. Buoyancy estimation for the live trained birds based on their measured body mass using body density estimates derived from carcasses (mandarins) and the literature (pochards). Bold numbers correspond to the mean (±SD) body density used in the calculations and the resulting mean buoyancy of our birds.

Ducks	Birds measured	Body mass (gr)	Volume (ml)	Density (kgm^{-3})	Buoyancy (N)	Species
Pochards	Lovvorn & Jones (1991)	1104	1591	693.9	4.8	*Aythya valisineria*
		924	1312	704.3	3.8	*Aythya americana*
		617	896	688.6	2.7	*Aythya affinis*
		680	983	691.8	3.0	*Aythya collaris*
				**694.6 ± 6.8**		
	This study	499	718	694.6	2.1	*Aythya nyroca*
		499	718	694.6	2.1	*Aythya nyroca*
		508	731	694.6	2.2	*Aythya nyroca*
		519	746	694.6	2.2	*Aythya nyroca*
		567	816	694.6	2.4	*Aythya nyroca*
					**2.2 ± 0.11**	
Mandarins	Carcasses	538	750	717	2.08	*Aix galericulata*
		357	569	628	2.07	*Aix galericulata*
				**672**		
	Lovvorn & Jones (1991)	559 ± 45	788 ± 64	709		*Aix sponsa*
	This study	730	1086	672	3.5	*Aix galericulata*
		570	848	672	2.7	*Aix galericulata*
		710	1056	672	3.4	*Aix galericulata*
		650	967	672	3.1	*Aix galericulata*
		540	803	672	2.6	*Aix galericulata*
					**3.1 ± 0.36**	

Journal of Experimental Biology • Supplementary information

Cited SI literature

Jacobs, E. N., & Sherman, A. (1939). Airfoil section characteristics as affected by variations of the Reynolds number. NACA report No. 586

Lighthill, M. J. (1970). Aquatic animal propulsion of high hydromechanical efficiency. *Journal of Fluid Mechanics*, *44*, 265–301.

Lovvorn, J. R., & Jones, D. R. (1991). Body mass, volume, and buoyancy of some aquatic birds, and their relation to locomotor strategies. Canadian journal of Zoology, 69, 288-2892. https://doi.org/10.1139/z91-407

Ribak, G., Weihs, D., & Arad, Z. (2004). How do cormorants counter buoyancy during submerged swimming ? *Journal of Experimental Biolgy*, 2101–2114.

Ribak, G., & Gurka, R. (2023). The hydrodynamic performance of duck feet for submerged swimming resembles oars rather than delta-wings. *Scientific Reports*, *13*, 16217. https://doi.org/10.1038/s41598-023-42784-w

Urca, T., & Ribak, G. (2021). The relationship between body size and flight power output in the Mango stem borer (*Batocera rufomaculata*). *Journal of Insect Physiology*, *133*, 104290.

Journal of Experimental Biology • Supplementary information

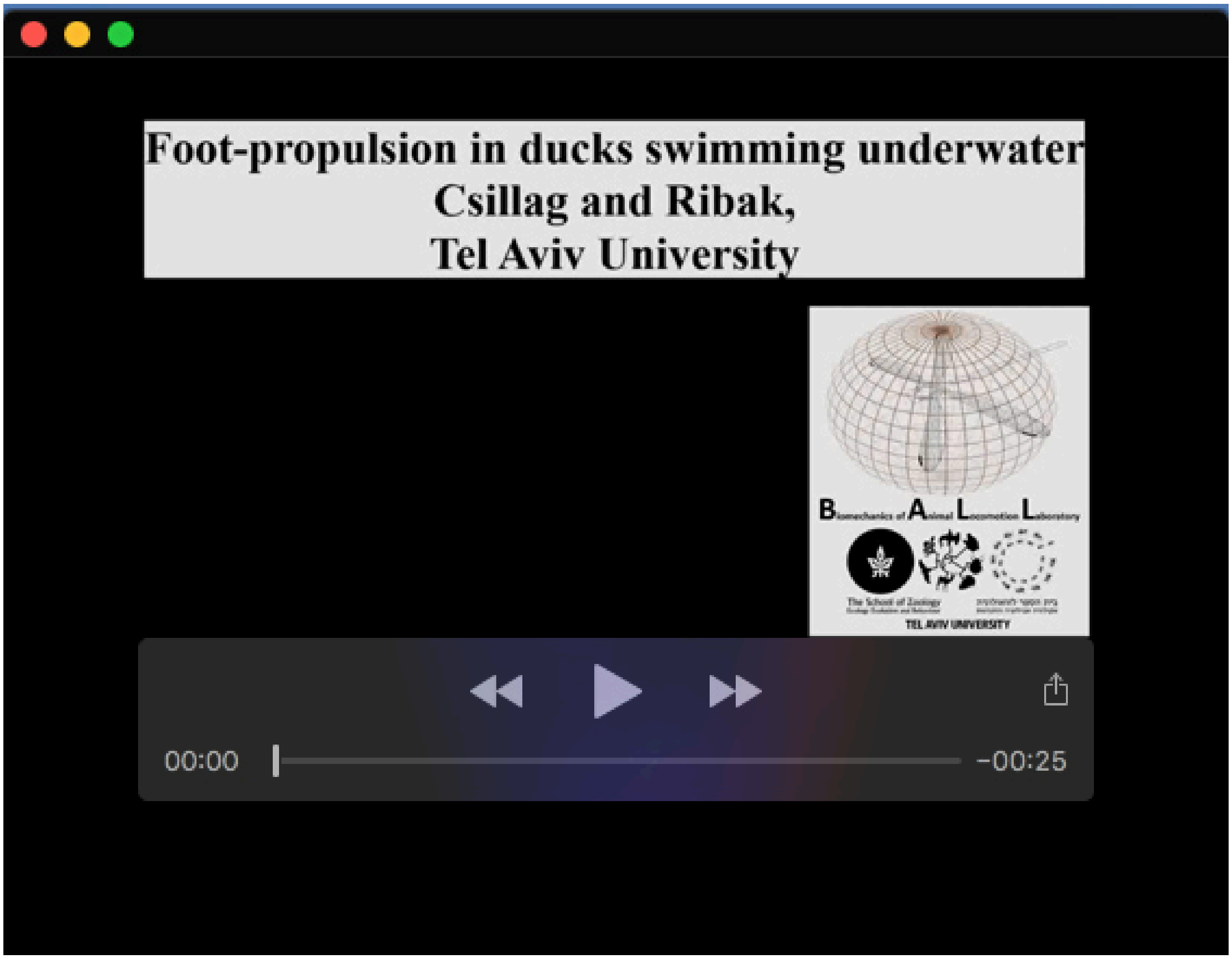

Movie 1. The high-speed movie clip shows an example of a mandarin duck swimming horizontally underwater followed by an example of a pochard swimming horizontally underwater. Such movies from two cameras were used to analyze the paddling kinematics. Next, the clip shows a female mandarin duck swimming horizontally underwater using wing propulsion. This duck was an exception that used its folded wings for wing propulsion consistently in the experiments. Consequently, it was excluded from the study that focused on the foot-propulsion. The end of the clip exemplifies the digitized points on the foot used to analyze the kinematics.

the average 3D motion of the foot during the power phase

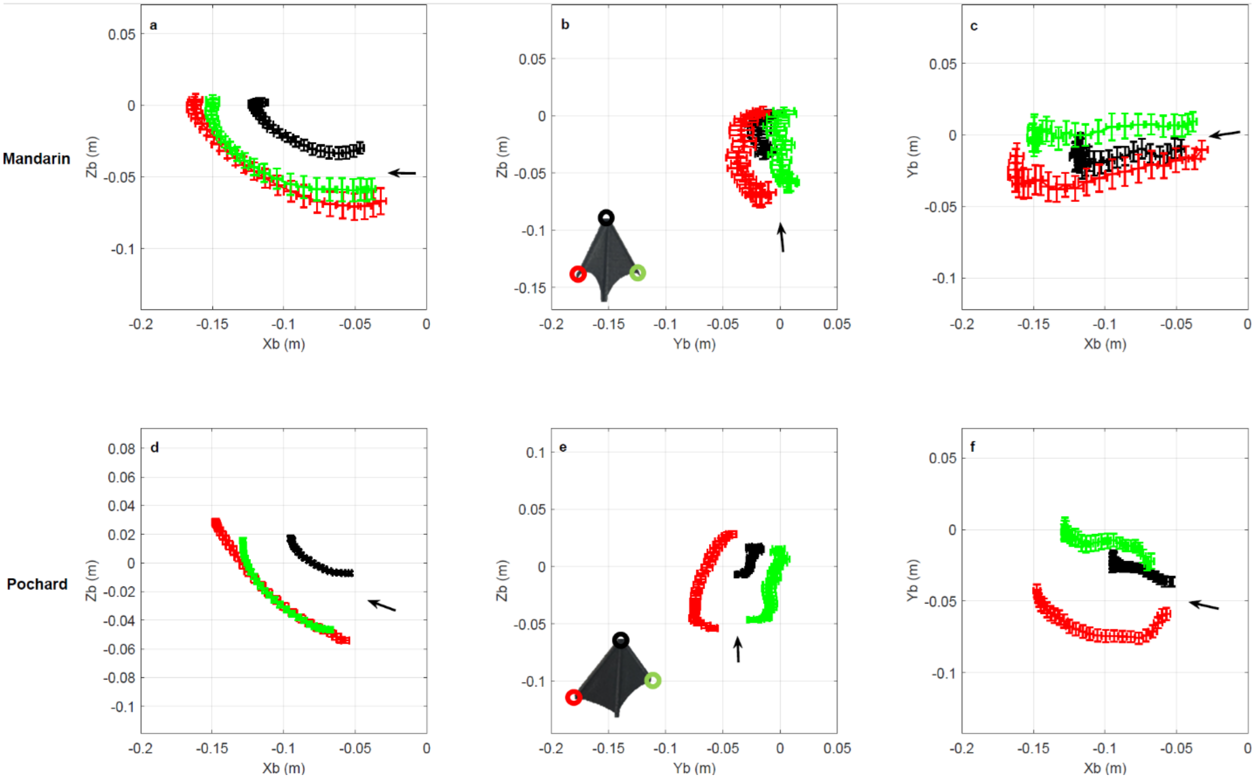

Fig. S3. The mean 3D (± SE) positions of points 5, 6, and 8 on the right foot (black, red, and green, respectively, see inserts in b and d) are shown at every 4% of the power phase duration. Columns correspond to the XbZb (a,d), YbZb (b,e) and XbYb (c,f) planes. The upper panel (a-c) denote the average foot motion of the mandarins and the lower panel (d-f) denotes the foot of the pochards. Black arrow show the direction of progression of the foot from the power phase initiation. The positions are relative to the position of the center of mass (0,0,0), i.e. in a coordinate system moving with the swimming duck.

Journal of Experimental Biology • Supplementary information

Relationship between thrust and swimming speed

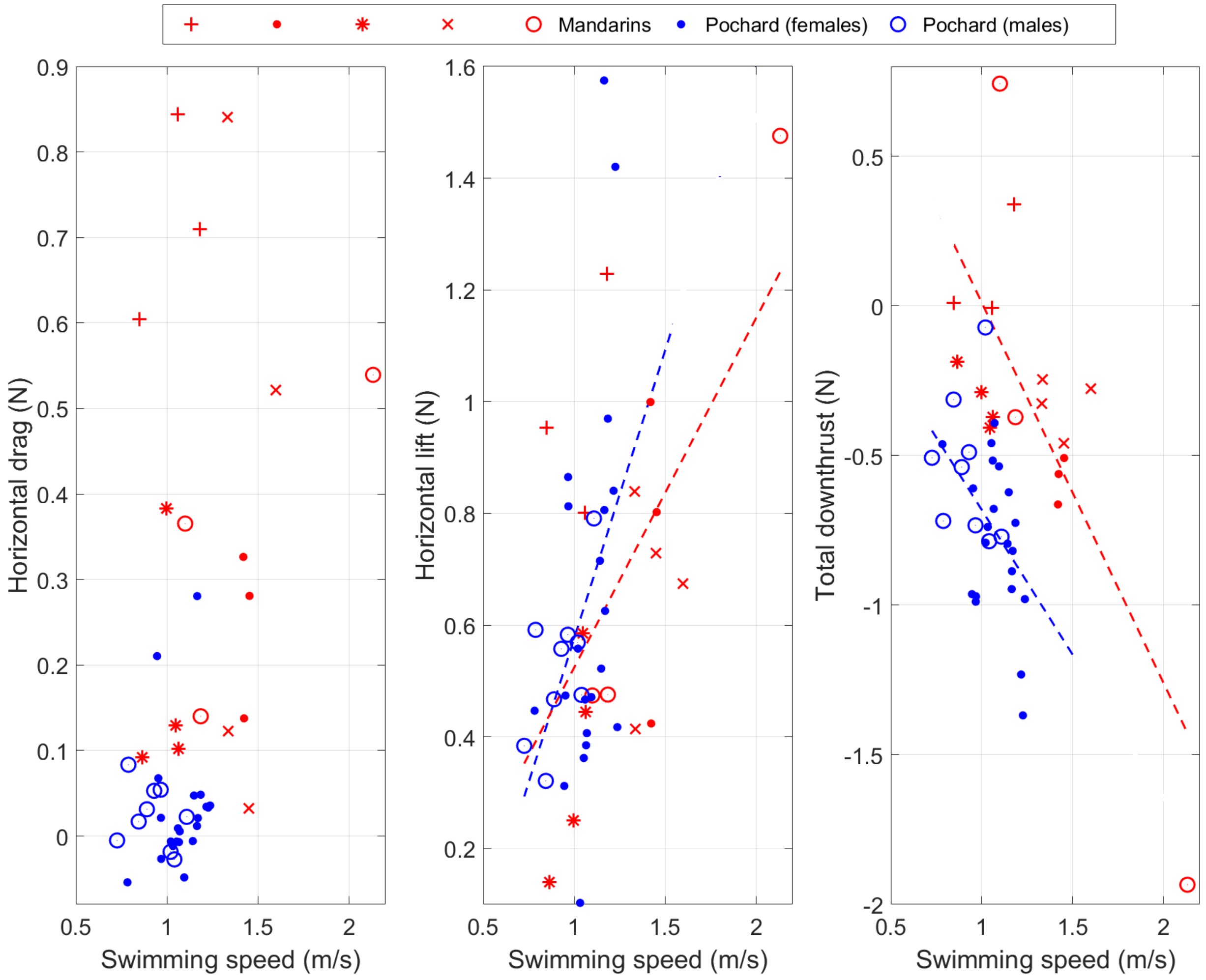

Fig. S4. The contribution of hydrodynamic drag (a) and lift (b) generated by the foot to the forward thrust of paddling ducks as a function of their swimming speed. Positive values imply thrust in the swimming direction. Negative values imply forces resisting the forward motion c) The relationship between swimming speed and total downthrust (downward directed propulsive force) generated by the foot during the power phase. Negative values are directed downward and resist the buoyancy of the birds. Positive values propel the bird upwards. Each dot represents the mean force calculated from the power phase of a single power phase where mandarins are depicted by red symbols and pochards by blue symbols. Full and empty symbols denote female and male pochards, respectively, and different red symbols correspond to different birds in the study.

Journal of Experimental Biology • Supplementary information

The paddling gait of the ducks while swimming on the surface

The same ducks were filmed swimming at the water surface and their feet were scored in the movies as described for submerged swimming in Fig. 1b of the main text. On the water surface both duck species used alternate paddling consistently (Fig. S4). The duty cycle, phase sift and recovery overlap were not correlated with the swimming speed on the surface ($R<0.52$ and $p>0.056$ in all cases). Compared to surface swimming pochards changed their gait underwater while mandarins didn't. The phase shift duty cycle and recovery overlap did not differ between surface and submerged swimming in mandarins (GLM, $p>0.6$ for all three parameters). Pochards, decreased their phase shift (Mann-Whitney, $p=0.007$) and increased the recovery overlap (Mann-Whitney, $p<0.001$) during submerged swimming compared to surface swimming. Their duty cycle remained the same on and below the water surface (GLM, $p=0.063$).

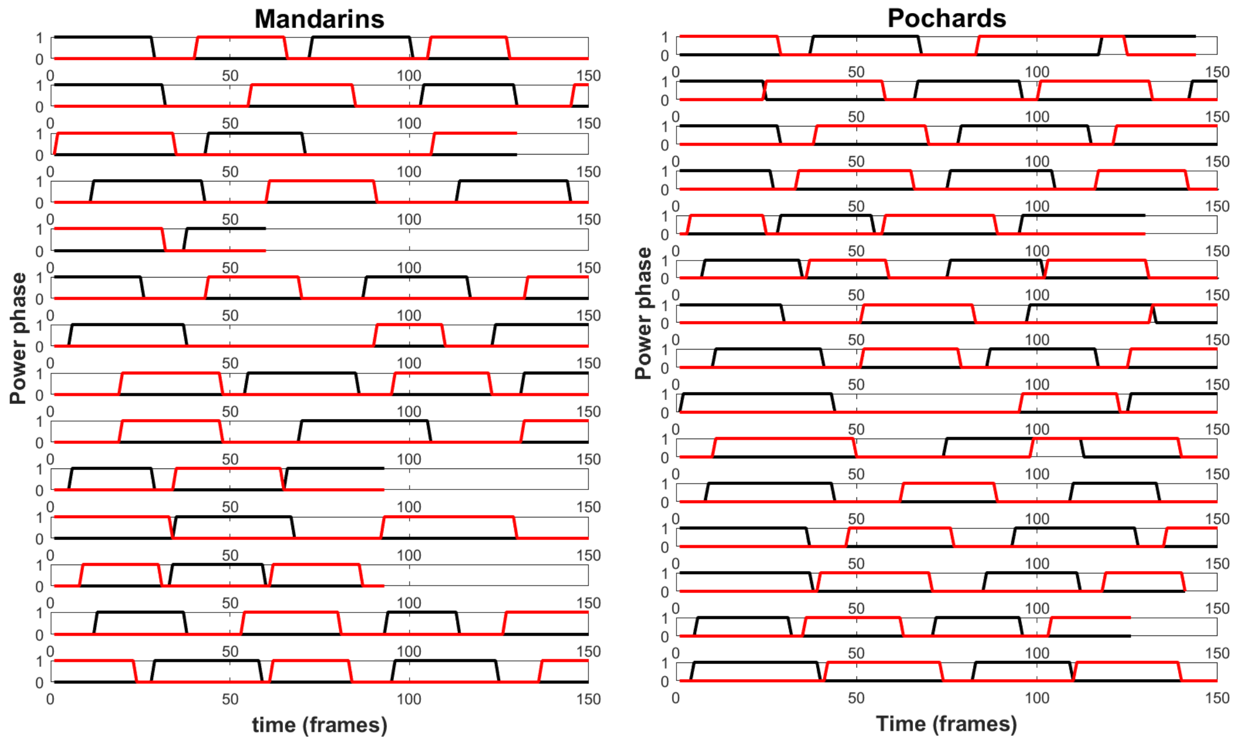

Fig. S5. Both species of ducks use alternate paddling while swimming on the surface. Each row represents a single movie of surface swimming, in which each frame was scored as 1 when the foot was engaged in a power phase and 0 when it was not. Red and black lines denote the scores for the contralateral feet as a function of time. The ducks are the same birds studied for their underwater swimming. Data from 15 randomly selected movies are shown for each duck species.

Journal of Experimental Biology • Supplementary information
